# Transcription Factors Exhibit Differential Conservation in Bacteria with Reduced Genomes

**DOI:** 10.1371/journal.pone.0146901

**Published:** 2016-01-14

**Authors:** Edgardo Galán-Vásquez, Ismael Sánchez-Osorio, Agustino Martínez-Antonio

**Affiliations:** Center for Research and Advanced Studies of the National Polytechnic Institute, Campus Irapuato, Genetic Engineering Department, Cinvestav, Km. 9.6 Libramiento Norte Carr. Irapuato-León 36821, Irapuato Gto, México; University of Lausanne, SWITZERLAND

## Abstract

The description of transcriptional regulatory networks has been pivotal in the understanding of operating principles under which organisms respond and adapt to varying conditions. While the study of the topology and dynamics of these networks has been the subject of considerable work, the investigation of the evolution of their topology, as a result of the adaptation of organisms to different environmental conditions, has received little attention. In this work, we study the evolution of transcriptional regulatory networks in bacteria from a genome reduction perspective, which manifests itself as the loss of genes at different degrees. We used the transcriptional regulatory network of *Escherichia coli* as a reference to compare 113 smaller, phylogenetically-related *γ*-proteobacteria, including 19 genomes of symbionts. We found that the type of regulatory action exerted by transcription factors, as genomes get progressively smaller, correlates well with their degree of conservation, with dual regulators being more conserved than repressors and activators in conditions of extreme reduction. In addition, we found that the preponderant conservation of dual regulators might be due to their role as both global regulators and nucleoid-associated proteins. We summarize our results in a conceptual model of how each TF type is gradually lost as genomes become smaller and give a rationale for the order in which this phenomenon occurs.

## Introduction

Transcription is an essential molecular process through which cells respond and adapt to changing environmental conditions, such as different kinds of stress and nutrient deficiencies [1]. The regulation of this dynamic process is usually carried-out by proteins called transcription factors (TFs), which bind to upstream regions (called promoters) of target genes (TGs) and promote or inhibit the synthesis of RNA molecules (and the subsequent production of proteins). TFs can be classified as activators, repressors, or dual regulators according to whether they promote, prevent, or exert both regulatory actions on the transcription of genes, respectively [2]. Although other molecular regulatory mechanisms might be operating at other stages of the gene expression process, such as DNA-methylation, RNA interference, etc., transcriptional regulation mediated by TFs is the predominant type of control in gene expression [3].

When the product of a regulated gene is a TF that regulates its own expression or the expression of other genes, the resulting regulatory interactions and the corresponding genes can be conceptualized as edges and nodes in a transcriptional regulatory network (TRN) whose topology dictates a hierarchy between regulators and target genes [4]. According to the number of genes they regulate, TFs can be ranked and hence defined as global regulators, when they act as hubs (highly connected nodes) in a network; and local regulators, when they regulate few genes, usually an operon or genes represented as terminal nodes in a regulatory network [5].

TRNs organize the responses of organisms to particular conditions and, despite their diversity, they share several topological features, including conserved network motifs, similar hierarchies, and scale-free structures [4, 6, 7]. These general patterns have been uncovered thanks to the availability of whole genome sequences of several organisms and to advances in detailed experimental techniques for the detection of protein-DNA interactions. In spite of the fact that network topologies obtained from computational methods alone are incomplete (or may contain links not supported to date by physical evidence) [8], analyses conducted mainly in free-living bacteria have revealed that their elements tend to change considerably, with the set of nodes corresponding to regulatory genes undergoing radical changes compared with the non-regulatory ones, which are more conserved among genomes [9–11].

On the one hand, the above alterations in the elements of a network may involve mutations that occur at a genome level, such as single nucleotide substitutions or those produced by the action of transposons, which affect one or a few nucleotides and can lead to the creation or deletion of DNA-binding sites on promoter regions [12]. On the other hand, network alterations also include changes that arise from gene duplication or horizontal gene transfer events that add large DNA fragments (containing one or more genes) to the genomes [13, 14]. Through these evolution-driven processes some genes and interactions are gained while others are lost. The appraisal of this phenomenon raises some intriguing questions concerning the evolution of TRNs (*i*.*e*. the gain or loss of nodes and interactions in a network throughout time) [14]. For example: How does the structure of TRNs evolve in different organisms? Is there a tendency in the organization of regulatory networks for organisms undergoing massive loss of their genes? Is a particular type of regulatory gene favored during evolution such that the regulators they code for are more conserved than others? If so, what cellular functions are they regulating?

Some of the above questions have been partially addressed by studying the TRNs of model organisms, revealing interesting facts. For example, Teichmann and Babu [15] found that more than two-thirds of the known interactions in the TRNs of *E*. *coli* and *Saccharomyces cerevisiae* evolved as a consequence of gene duplication. Over one-half of these regulatory interactions were inherited from ancestral duplications of TFs and target genes. Only a small portion of the remaining fraction of regulatory interactions (not evolved by duplication) evolved by gene recombination or innovation. In the same line, Lagomarsino *et al*. [16] discovered that horizontally transferred genes (which impact on network growth) mostly contribute to cellular fitness under very particular conditions, but they are not essential in unstressful environments. These genes generally locate at the bottom of the network hierarchy in the TRN of *E*. *coli*. In another related work, Molina and van Nimwegen [17] studied how the average number of regulatory sites per intergenic region and the average number of sites regulated by a particular TF vary with genome size (across 105 free-living bacteria). They found that the structure of TRNs varies substantially with genome size. More precisely, they concluded that small genomes code for few TFs (each binding to a large number of target sites) while large genomes contain many TFs (each binding to a few sites), in which case TFs seem to be more specialized.

While relevant for the understanding of network evolution, the studies described previously have not considered the loss of nodes or interactions in regulatory networks, perhaps due to the scarcity of whole genome data (particularly that of endosymbionts and obligate pathogens) until the last decade. In this work, we investigated the evolution of TRNs by considering organisms undergoing loss of their genes, with a particular interest in the way in which genes involved in regulatory functions are lost according to their type. As it is not trivial to determine from the genome size alone whether an organism has undergone reduction or growth of its genome, we selected as the subject of study those bacteria in the *γ*-proteobacteria class with genomes smaller than that of *E*. *coli*, 19 of which are thought to have evolved into obligate pathogens and endosymbionts. These 19 organisms, which are restricted to live inside other organisms [18], are believed to have been subjected to a Muller’s ratchet process, a phenomenon that results in the accumulation of slightly deleterious mutations [19]. These mutations in turn bring about genomes with low G+C content, accelerated rate of nucleotide substitution, and loss of adaptive codon bias [19, 20].

*E*. *coli* is phylogenetically related to endosymbiotic *γ*-proteobacteria. This is an advantage for our purposes, since the TRN of *E*. *coli* is the most studied and well characterized among all bacteria. Hence, we used this regulatory network as a template to reconstruct the networks of the bacteria studied in this work. We followed a comparative genomics approach to identify conserved elements in the networks of the different organisms and applied a combination of correlation analysis with phylogenetically independent contrasts [21] to correct for statistical dependencies induced by phylogenetic relationships (see Methods, section 2). The picture that have emerged from our study is that there is an order in the way TFs are conserved: on the one hand, activators (which represent the major proportion of TFs in large genomes), followed by repressors, become less abundant as genome size decreases, eventually disappearing in conditions of extreme genome reduction; on the other hand, dual regulators become more preponderant as genomes get smaller, being the last TF type lost under extreme genome reduction. Moreover, we observed that such TFs are global regulators and some of them are nucleoid-associated proteins (NAPs) involved in the control of nucleoid structure. All our findings were integrated into a conceptual model that portraits network evolution in organisms undergoing genome reduction and describe the order in which this phenomenon happens. Finally, we highlight the essential ideas arising from our study and discuss their implications in the understanding of gene regulation in reduced genomes.

## Methods and Data

To study the evolution of TRNs from the perspective of reduction, we selected a set of 113 genomes belonging to organisms phylogenetically related to *E*. *coli* and reconstructed the corresponding transcriptional networks using a comparative genomics approach (section 2.1). After assessing the preferential conservation of TFs in terms of their regulatory nature, by formulating hypothesis tests for each type of TF in the 113 genomes (as described in section 2.2), we applied the method of phylogenetic contrasts (see section 2.3) to correct for dependencies in our data and calculate correlations between each type of regulation (*i*.*e*., activation, repression, and dual regulation) and genome size. To account for the influence of other variables in the observed correlations, we considered global regulators -which we had to first identify using a metric developed in a previous work (see section 2.4)- and NAPs as additional factors. We subsequently performed multivariate linear regressions, defining as criterion variables the relative fractions of each TF type, and as predictors the remaining variables of interest, specifically: genome size, global nature of regulators, and their function as NAPs (section 2.5). From the regression coefficients obtained in the latter analysis, we could infer the contribution of each factor of interest on the conservation of each type of regulator.

### 2.1 Reconstruction of Transcriptional Regulatory Networks

We selected a set of 113 genomes of *γ*-proteobacteria whose sizes range from 0.159 Mbp (corresponding to *Candidatus Carsonella Ruddii PV*) to 4.639 Mbp (corresponding to *E*. *coli K-12 MG1655*), trying as much as possible to reduce the redundancy of genomes that belong to a same species and keeping the difference in genome size between pairs of contiguous genomes below 400 Kbp. From these 113 genomes, which are smaller than the genome of *E*. *coli*, 19 belong to symbionts. Information on complete genome sequences was downloaded from the NCBI genomes website [22]. The TRNs of the 113 genomes were reconstructed by the conventional Regulog approach [23]. This comparative genomics technique aims at finding conserved transcriptional regulatory interactions in organisms where knowledge of their TRNs is missing, using for this purpose information about the interactions in a known (reference) regulatory network. Regulog infers regulatory interactions based on the assumption that orthologous TFs generally regulate the transcription of orthologous target genes (TGs). For example, if a TF and its TG in *E*. *coli* have orthologous *TF’* and *TG’* in other organism, then a regulatory interaction is inferred as conserved in the target genome [23]. Thus, using the well-characterized transcriptional network of *E*. *coli* as a template (composed of 1,784 nodes and 4,058 interactions between TFs and their TGs) [24], we identified orthologous TFs and TGs in each target organism via the classic bidirectional best-hit method [25] (see S1 Table).

**Fig 1:**
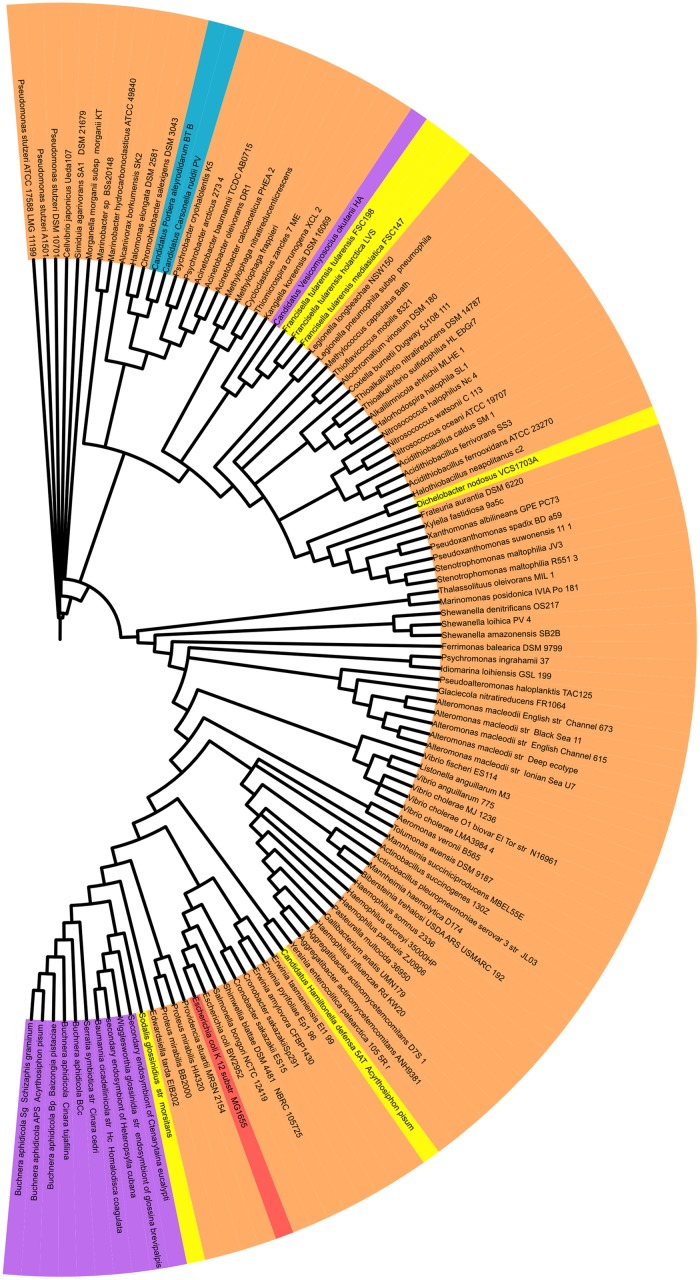
Phylogeny of the *γ*-proteobacteria studied in this work. The tree lists the 113 bacteria considered in this study. Free-living bacteria are in orange, host-restricted symbionts and pathogens in yellow, obligate symbionts and pathogens in violet, tiny-genomes symbionts in blue, and *E*. *coli K-12 MG1655* in red. The phylogeny was constructed using the software MrBayes-3.2 based on the 16S rRNA genes.

For each protein in the transcriptional network of *E*. *coli*, we did a Blast search against the genome of each of the 113 *γ*-proteobacteria selected in this study (Fig 1). The best hit obtained (for each genome) was in turn used as a query sequence in a Blast search against the genome of *E*. *coli*. We define orthologous proteins when the best hit in the last step (when the search parameters are reversed) corresponded to the protein in the genome of *E*. *coli* originally used in the first query. Orthologs were accepted if they had an *e*-value <1*e*-6, sequence identity >30%, and alignment length >60% of the individual proteins.

Our approach to the reconstruction of TRNs involves two basic assumptions. The first is that TFs retain their type of regulatory function (*i*.*e*., activation, repression and dual regulation) throughout all the studied genomes, and that the corresponding TGs conserve their TF binding sites. The second assumption is that if the sequences corresponding to TFs and TGs are conserved, then regulatory interactions are conserved too. As a consequence, the impact those mutations of genes and promoter sequences have on the regulatory interactions are not considered. The fact that the 113 genomes compared in our analysis belong to closely related organisms makes our network inferences more reliable, as we circumvent to a certain extent some of the limitations inherent in our method of reconstruction; however, the results and conclusions derived from this method are valid to the extent that the underlying assumptions do not contradict experimental evidence. For example, it is known that the assumptions described above become inadequate as the evolutionary distance increases and that some orthologs with high sequence similarity may not be functionally conserved, in which case the reconstruction approach would be generating false positives.

The 113 genomes were divided into four categories as defined by Moran’s group [18]: the first included bacteria classified as free-living, with genomes smaller than that of *E*. *coli*; the second contains bacteria identified as host-restricted endosymbionts or pathogens; the third contains obligate pathogens or endosymbionts; and the fourth contains endosymbionts exhibiting extreme genome reduction (see S2 Table). Additionally, from information obtained from EcoCyc [26], 190 TFs out of the 196 known to regulate at least one gene in the TRN of *E*. *coli* were classified as activators, repressors, or dual regulators, according to the type of net effective regulation they exert over their TGs (6 of these TFs do not have information about their type of regulatory action). We also classified and determined the biological functions of all the orthologous genes, found in previous steps, using the parental categories of Gene Ontology [27, 28], (see S3 Table).

### 2.2 Analysis of Transcription Factor Conservation

To decide if reduction of genome size favors the conservation of a particular type of TF (*i*.*e*., activator, repressor, or dual regulator), we performed statistical tests for each of the 113 studied genomes, defining as null hypotheses those statements indicating a reduction or no variation in the relative genome fraction of each type of regulator with respect to the corresponding fraction in the genome of *E*. *coli*. Accordingly, the alternative hypotheses were formulated as the logical complements to such statements. More precisely, the general form of the tests for the case of activators is as follows:

$$H_{0|i}^{a}:f_{i}^{a}\leq f_{r}^{a}$$

$$H_{1|i}^{a}:f_{i}^{a}>f_{r}^{a}$$

Where $H_{0|i}^{a}$ (with the index *i* ranging from the 1^st^ to 113^th^ genome) denotes the null hypothesis that the fraction of activators in the *i*^th^ genome ($f_{i}^{a}$) is less or equal to the corresponding fraction of activators in the reference genome ($f_{r}^{a}$). An identical form of the test applies to repressors and dual regulators. When the null hypotheses are rejected, the corresponding alternative hypotheses ($H_{1|i}^{a}$,$H_{1|i}^{r}$ or $H_{1|i}^{d}$ 1≤*i*≤113, as the case may be) -that there is in fact a preferential conservation of a particular type of regulator- are supported. If, on the contrary, the relative fraction of a regulator in a genome *i* is not significantly higher than the fraction in the genome of *E*. *coli*, then we fail to reject the corresponding null hypothesis and, in consequence, failed to support the alternative hypothesis.

To compute the *p*-values and determine the statistical significance (at the 0.05 level) of the tests, we used a hypergeometric distribution for each kind of TF in the 113 genomes. The reason for this is that such distribution describes the probability that given a reference genome (in this case that of *E*. *coli*) coding for *N* TFs in total, from which *K* TFs are of a particular type (activator, repressor, or dual regulator), *k* elements from the latter appear in another genome that contains *n* orthologous. We generated three hypergeometric distributions for each genome used in this study (one for each type of regulation) with defining parameters *n* and *k*; where *n*, representing the number of orthologous TFs, is fixed in each genome and *k*, which varies from 0 to *n*, is the number of TFs of the particular regulatory type (activator, repressor, or dual regulator), which corresponds to a relative genome fraction whose probability of occurrence we want to compute. The processing of data and computations of parameters were performed in R (see S4 Table) [29].

To assess an overall statistical significance from all the 113 tests performed for each type of TF, we combined the *p*-values obtained from the set of independent tests corresponding to activators, repressors, and dual regulators, respectively, using Fisher’s method [30, 31]. A requirement for the application of this kind of meta-analysis is that the same undelaying form of the hypothesis test is used in the independent, more elementary tests, which is valid for the present study.

### 2.3 Analysis of the Influence of Phylogenetic Relationships

To discern how the genome fraction of each type of regulator (*i*.*e*., activator, repressor, dual) varies with the reduction in genome size, we calculated the Pearson correlation coefficient, which measures the degree and direction of linear relationships between these variables. Because the genomes being studied belong to closely related organisms that are part of a hierarchically structured phylogeny, our data points cannot be considered as statistically independent from one another. This dependence (caused by phylogenetic similarity) may generate significant correlations between the number of TFs and genome size when no causal link exists between them. To correct for such effects, we incorporated phylogenetic information into our statistical analysis using Felsentein’s independent contrasts [21]. This method computes weighted differences (called contrasts) between the trait values associated to pairs of nodes at each bifurcation point in a known phylogeny, assuming evolution follows a random walk pattern in time. The resulting contrasts are, in principle, independent and normally distributed, and thus can be used in conventional statistical analyses. A detailed account of the method can be found in the references [21, 32, 33].

We used the PDAP:PDTREE module [33, 34] of Mesquite software version 3.02 [35] to calculate standardized phylogenetically independent contrasts for the following variables: genome size, frequency of activators, frequency of repressors, frequency of dual regulators, fraction of activators, fraction of repressors, fraction of dual regulators, fraction of global regulators and fraction of NAPs (see S5 Table), and subsequently determined the correlations and regressions through the origin. This approach demands that contrasts in the X-axis be “positivized” and that the original dataset has a normal distribution [32], a requirement that has been met in this work. The phylogenetic tree necessary for the calculation of contrasts was constructed with the program MrBayes-3.2, which implements a Bayesian inference method [36, 37]. To construct the tree, we employed the 16s ribosomal gene sequences of all the 114 bacterial genomes (including *E*. *coli*) used in this study and performed 2 runs with 1x10^7^ generations, a 25% burn-in, and sampling every 1000^th^ iteration.

After calculating the phylogenetic contrasts, as described in the above paragraphs, we performed significance tests using a *p*-value of 0.05 to determine whether the obtained correlations between the calculated contrasts could be attributed to chance. Because the correlations turned out to be significant, we accepted the correlations as measuring statistically dependent variation and showing an underlying relationship between the variables.

### 2.4 Identification of Global Regulators

TRNs contain highly connected nodes called global regulators or regulatory hubs, which contribute to the structural cohesion and robustness of the networks and to their functional coherence [38]. In a previous work [5], we described the operational criteria for the identification of a global regulator, which included: i) number of regulated TGs; ii) number of TFs to which it co-regulates, iii) number of coupled sigma factors in the regulation of TGs; and iv) number of TFs that it regulates. From the definitions of these parameters, we proposed in Galan E., *et al*. [39] an equation (see Eq 1) that associates, to every transcription factor *x*, a value *G*(*x*)∈[0,1] indicating its global activity in the context of a regulatory network:

$$\text{G}\left(\text{x}\right)=\frac{1}{4}\left(\frac{\text{TFR}\left(\text{x}\right)}{{\text{N}}_{\text{TF}}+{\text{N}}_{\text{SF}}-1}+\frac{\text{GR}\left(\text{x}\right)}{{\text{N}}_{\text{G}}}+\frac{\text{SF}\left(\text{x}\right)}{{\text{N}}_{\text{SF}}}+\frac{\text{CR}\left(\text{x}\right)}{{\text{N}}_{\text{TF}}-1}\right)$$ (1)

**Fig 2:**
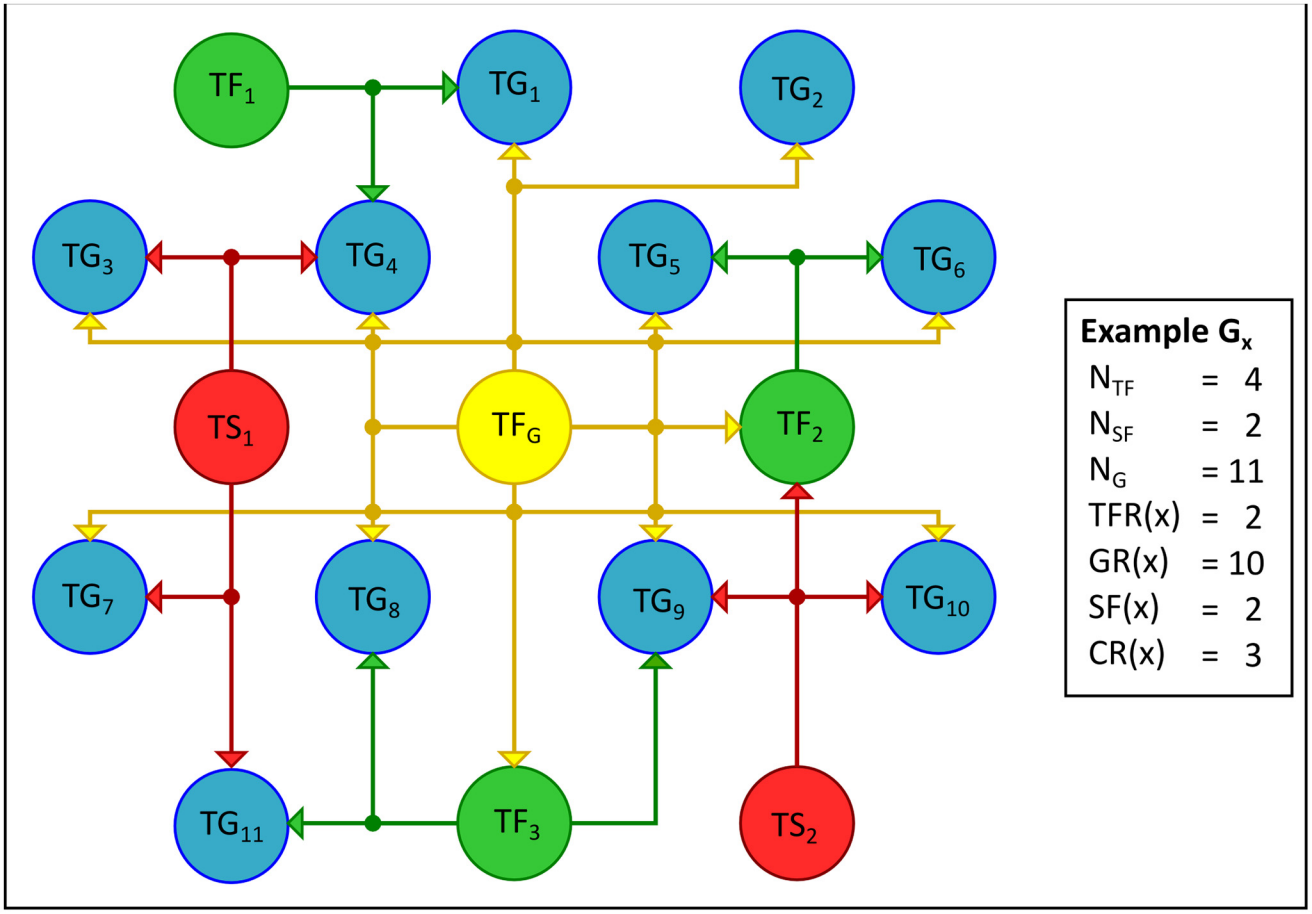
Identification of global regulators. This figure exemplifies the calculation of the metric for a global regulator TF_G_ (yellow node) in a hypothetical network consisting of 17 nodes and 26 interactions, in which TF_G_ regulates 10 TGs (blue nodes) and 2 TFs (green nodes); in addition, it co-regulates along with 2 sigma factors (red nodes) the nodes labeled as TG_3_, TG_4_, TG_7_, TG_9_, TG_10_, and TF_2_; and along with 3 TFs, the following target genes: TG_1_, TG_4_, TG_5_, TG_6_, TG_8_, and TG_9_. From the metric described by Eq (1), we obtain a value of G(TF_G_) = 0.8272.

Where *N*_*TF*_ is the number of TFs, *N*_*SF*_ the number of sigma factors, and *N*_*G*_ the number of non-regulatory genes in the network. On the other hand, *TFR*(*x*) represents the number of TFs regulated by *x*, *GR*(*x*) the number of non-regulatory genes regulated by *x*, *SF*(*x*) the number of sigma factors required by *x* to regulate its TGs, and finally, *CR*(*x*) the number of TFs co-regulating with *x* other TGs. The metric described by Eq (1) defines a hierarchy of global regulators in the TRN of *E*. *coli* and permits the identification of conserved global regulators in each of the reconstructed TRNs (see Fig 2 for an example).

### 2.5 Multivariate Regression Analysis

As the conservation of the fractions of each TF type may be influenced not only by genome size but also by the global nature of regulators, as well as the fact that they act as NAPs, we assessed the effects of these additional variables by performing multivariate linear regressions. In these equations we defined as dependent variables the relative fractions of each TF type (also known as criterion variables), and include as independent variables (or predictors) the factors whose influence we want to measure; specifically, genome size, global nature of regulators, and their function as NAPs. The partial regression coefficients obtained from the multiple regressions allowed us to discern the contribution of each variable of interest on the conservation of each type of regulator when the effect of the remaining predictors is held constant.

In summary, we found the coefficients (*β*_0,_*β*_1,_*β*_2,_*β*_3_) of the multivariate Eq (2), using a built-in R routine implementing a least squares method.

$$Y_{TF}=\beta_{0}+\beta_{1}X_{1}+\beta_{2}X_{2}+\beta_{3}X_{3}+\varepsilon$$ (2)

*Y*_*TF*_ is the least squares prediction of the relative fraction corresponding to a particular type of TF and the variables *X*_1_, *X*_2_, *X*_*3*_ represent the predictors (genome size, global regulators, and function as NAPs, respectively) whose contribution to the criterion variable, *Y*_*TF*_, is addressed in this analysis. The error *ε* is assumed to have a normal distribution with mean 0 and constant variance σ^2^. *β*_*0*_ is the *Y* intercept of the hyperplane defined by Eq (2) and is interpreted as the predicted value of *Y*_*TF*_ when all the predictors are zero. The other partial regression coefficients (*β*_1,_*β*_2,_*β*_3_) are the corresponding slopes of *Y*_*TF*_ on each of the predictors, keeping the remaining variables fixed [40]. Being coefficients expressed in different units, the *β*’s cannot be directly used to compare the contribution of each predictor on the value of *Y*_*TF*_. To circumvent this problem, we transformed the independent variables (*X*_1_, *X*_2_, *X*_*3*_) into standard deviates or “*Z* measures” and obtained the corresponding standardized partial regression coefficients (*b*_1_, *b*_2_, *b*_3_).

After transforming the *β*’s into *b*’s, we used the relative magnitude of *b* coefficients (considering their *p*-values) to compare the strength of the relationship between *Y*_*TF*_ and each particular predictor, when all the other predictors in the regression equation are kept constant. Thus, we could infer which predictor had the strongest influence on the conservation of each type of regulator [41].

## 3 Results and Discussion

### 3.1 Conservation of Activators, Repressors and Dual Regulators

In this work, we studied the evolution of TRNs in bacteria taking as a reference the regulatory network of *E*. *coli*. From this network, we reconstructed 113 TRNs of *γ*-proteobacteria with genomes smaller than that of *E*. *coli*. This was done following a comparative genomics approach (as explained in section 2.1). The studied bacteria included 19 obligate pathogens and endosymbionts, which are convenient biological models for the study of regulatory network evolution in the context of extreme genome reduction (Fig 1). We classified 190 transcription factors in the TRN of *E*. *coli* according to their regulatory activity into activators, repressors, and dual regulators. The presence (or absence) of these regulators was identified in all of the 113 γ-proteobacteria.

To measure the degree of correlation between the type of regulation and genome size, we corrected for phylogenetic dependencies in our data and performed statistical correlation analyses as described in section 2.3. We obtained a coefficient of *r* = 0.56 with *p*-value = 1.53e-10, for the correlation between the number of activators and genome size; *r* = 0.71 with *p*-value = 2.2e-16, for the number of repressors and genome size; and *r* = 0.64 with *p*-value = 2.53e-14, for the number of dual TFs and genome size. These coefficients seemed to indicate a whole picture in which the numbers of TFs decrease as genome size become smaller, in agreement with one of the scaling laws found in [42]. However, since such correlation may be the result of a generalized, random gene loss, we performed significance tests (at the level of 0.05) on the relative fractions of TFs obtained from each of the 113 genomes and for each type of regulation (refer to section 2.2 for further details). After applying Fisher’s method [30, 31] (see section 2.2) to combine the resulting independent *p*-values for each type of regulator, the relative fractions corresponding to activators (*p*-value = 0.99) and repressors (*p*-value = 0.99) in the 113 genomes turned out to be not significant (at the 5% level). Nevertheless, the fraction of dual regulators produced a statistically significant result (*p*-value = 2.90e-50), which indicates that small genomes favor the conservation of dual TFs (see S4 Table).

**Fig 3:**
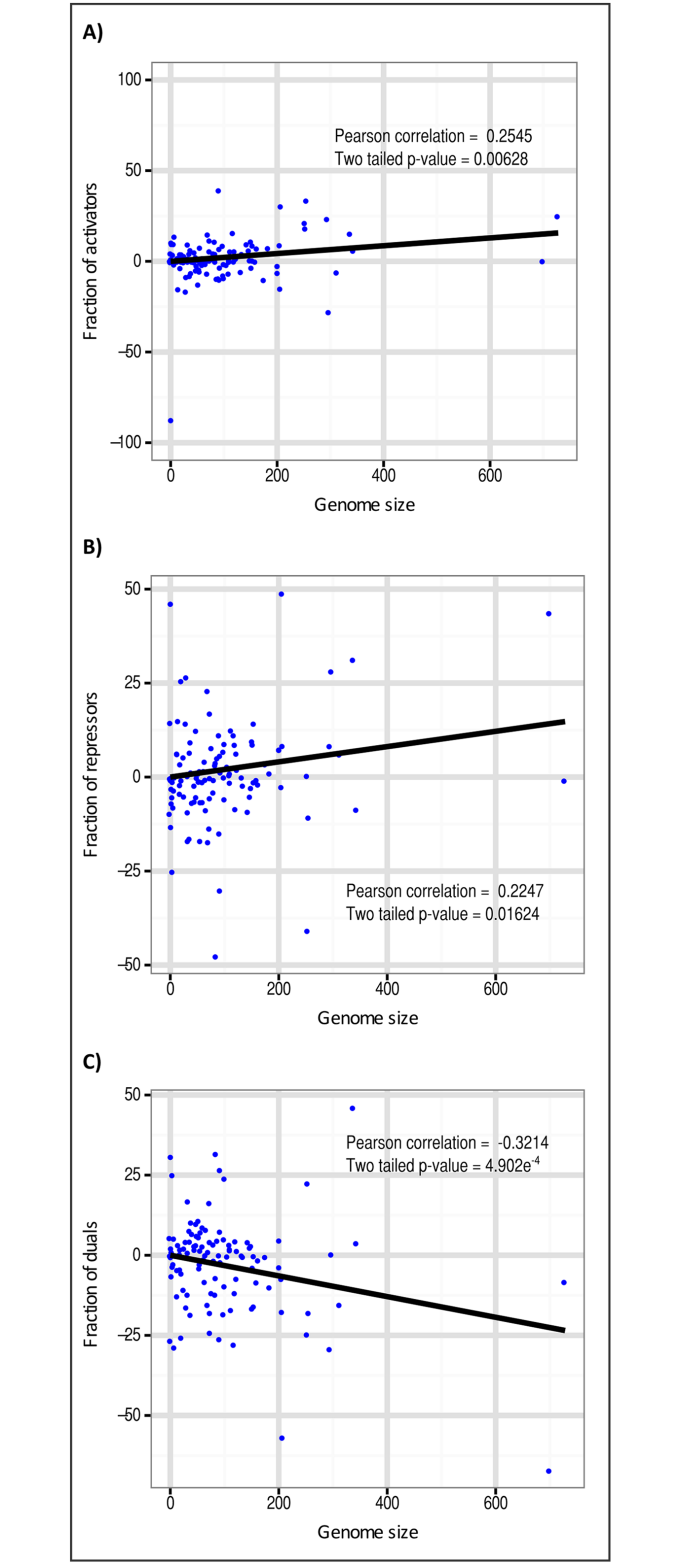
Phylogenetic contrasts of conserved transcription factors. Regression line through the origin and the Pearson correlation with corresponding *p*-value for A) Conservation of activators, B) Conservation of repressors and C) Conservation of dual regulators. Each point represents the contrast between the fraction of each TF type versus genome size (contrasts positivized as recommended by Garland [32]).

To further assess the preference in the conservation of dual TFs as genomes become smaller, a similar analysis to the one described in the previous paragraph was performed, but in this case using the relative frequencies corresponding to each TF type, instead of the net number of TFs per genome. We found positive correlations for activators and repressors with correlation coefficients of *r* = 0.26 (*p*-value = 6.28e-3) and *r* = 0.23 (*p*-value = 1.62e-2) respectively, and a negative correlation for dual regulators *r* = -0.32 (*p*-value = 4.90e-4) (see Fig 3). Our data and statistical analysis (refer to section 2.3), revealed that from the whole of TFs, activators and repressors tend to be present in a major proportion in large genomes (*e*.*g*., those corresponding to free-living organisms) while the fraction of dual regulators become more prevalent in small genomes (*e*.*g*., those belonging to obligate endosymbionts). This result suggests certain evolutionary preference in the conservation of TFs during the process of genome reduction, being dual TFs the type of regulator most preserved in genomes exhibiting advanced reduction.

Because we realized that genome size, global regulator nature, and function as NAP, are variables that may affect the conservation of TFs, we performed a multivariable linear regression analysis (as explained in detail in section 2.5) to identify which of these variables have the highest influence on the conservation of each TF type. From the standardized partial regression coefficients computed for each linear regression, we inferred the effect that each predictor has on the conservation of activators, repressors, and dual regulators, respectively. For the case of activators and repressors, we found that the influence of the predictor variables (*i*.*e*., genome size, global regulator nature, and function as NAP) was negligible. As can be observed in Table 1, the standardized partial regression coefficients in these cases were very small, with only one of them being significant. In contrast to these results, the standardized partial regression coefficients for dual regulators indicated that the global nature of such regulators (with a *b* = 0.49 and *p*-value = 2.00e-4) might explain their preponderant conservation.

**Table 1:** Standardized partial regression coefficients.

	Activators TFs		Repressors TFs		Dual TFs	
Independent variables	Coefficient *b*	*p*-value	Coefficient *b*	*p*-value	Coefficient *b*	*p*-value
Genome size	0.21	0.03	0.04	0.68	-0.15	0.10
Global regulators	-0.10	0.50	0.12	0.42	0.49	2.00e-4
Function as NAPs	-0.09	0.54	-0.09	0.55	-0.07	0.61

Interestingly, a major portion of conserved, dual, global regulators had a role as nucleoid organizing proteins in endosymbionts. When computing the Pearson correlation between global regulators and NAPs, we found a strong relationship (with a correlation coefficient equal to 0.75). Thus, we concluded that such variables were not strictly independent, but were in a sense redundant as they provided similar information. In fact, removing any of them (but not both) from the corresponding multivariable regression equation did not have a notable effect on the predicted value of the dependent variable (fraction of dual regulators).

### 3.2 Biological Interpretation of TF Conservation in the Context of Genome Reduction

The above findings have a biological explanation in the light of the functions associated to each kind of regulator. In the case of activators, which generally depend on co-inducers to respond to intra- or extracellular signals (such as adaptive response, antibiotics, virulence factors, etc.) [43–45], their loss might not substantially affect the expression of their TGs, because the non-restrictive structure of nucleoids in prokaryotes with small genomes facilitates the access of the transcriptional machinery to promoter regions, thus making basal transcription of genes possible [46]. In addition to this, recent studies have provided strong evidence that RNA polymerases spend most of their search time for a binding site (around 85% of the total time) nonspecifically bound to DNA [47], which gives a large probability that positively regulated genes may still exhibit some level of expression in spite of the loss of their activators.

In the case of repressors, we found that their proportion becomes increasingly larger than the fraction of activators as genomes get progressively smaller. The biological justification of this result might rely on the fact that the loss of repressors can lead to unnecessary expense of cellular resources due to basal expression of TGs. Besides, the conservation of transcriptional repression might give some slight biological advantage over activation because homeostatic control is usually implemented through negative regulation, a recurrent motif in metabolic and gene regulatory processes [48]. Moreover, it is known that negative auto-regulation (when appropriately tuned) can speed up responses in cellular systems [49, 50] and this dynamical response may contribute to the maintenance of cellular homeostasis regardless the physiological state of the organism [51].

Finally, the preponderance of dual TFs in small genomes might be explained from the fact that these regulators permit more flexible control (positive and negative) over their target genes. In addition, from our observations that dual TFs are usually global regulators and their regulated genes fall in more than one functional class [52, 53], we speculate that dual regulators confer to organisms the ability of maintaining cellular fitness with a minimal regulatory machinery.

The above conjectures can be framed into the demand theory of gene regulation proposed by Savageau [54]. This theory states that the type of regulation required for a gene is a function of the demand of the protein it encodes for during the life-cycle of bacteria; that is, if a protein is required most of the time, their encoding gene should be positively regulated; on the contrary, if a gene product is required only sporadically, their encoding gene should be negatively regulated [54]. In this context, the conservation of a particular type of regulation might be chosen to optimize the use of the regulator [55]. Thus, there should be a balance between the fitness cost and benefit of conserving a set of regulated (or unregulated) genes. We hypothesize that in endosymbiont bacteria this balance is influenced by the relatively stable environment or condition in which they live, which leads to differential loss of their TFs.

### 3.3 Conceptual Model of TF Conservation in Reduced Genomes

Endosymbiont bacteria exhibit genomic changes associated to the process of genome reduction, leading a free-living organism to acquire a host-restricted lifestyle [18, 56]. Studies conducted in organisms with reduced genomes have been possible until recently when genome sequences of many endosymbiont bacteria have been reported.

As a result of our comparative analysis using these organisms, we have given additional support to previous findings in free-living organisms of a tendency to retain more non-regulatory genes than genes coding for TFs. In a complementary manner, we have also discovered a tendency of dual regulators to be more conserved than activators and repressors in the last stages of genome reduction.

**Fig 4:**
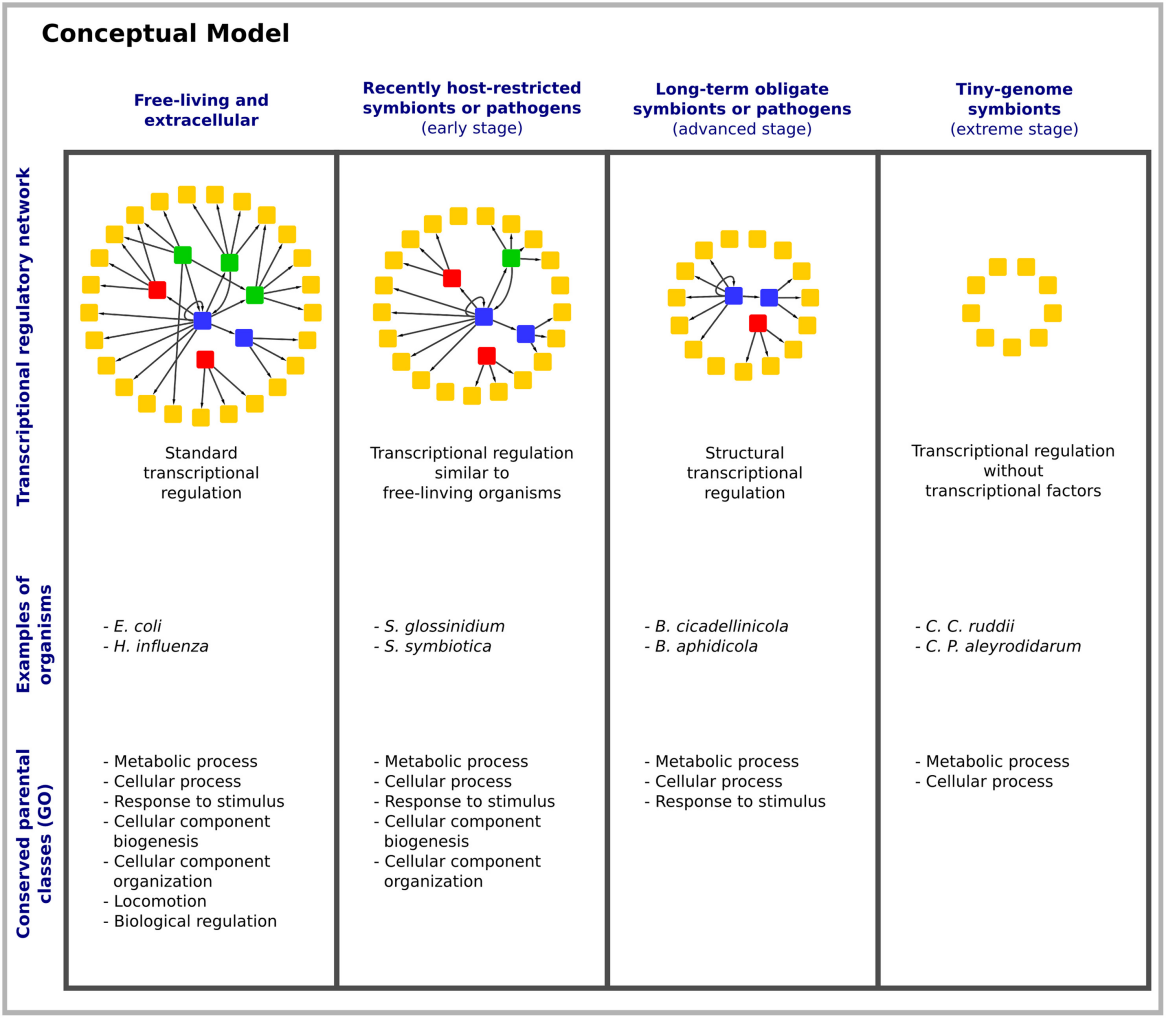
Conceptual model of transcriptional regulatory network evolution under the influence of genome reduction. For this model we used the classification proposed by N. Moran [18]. We start with a standard regulatory network (composed by activators, repressors, and dual regulators) corresponding to a free-living organism, in this case *E*. *coli*. In the first (early) stage of genome reduction, organisms are recently host-restricted symbionts or pathogens. These organisms have lost different TFs and TGs; however, the whole process of transcriptional regulation is similar to that of free-living organisms. In the second (advanced) stage of genome reduction, organisms turn into obligate symbionts or pathogens. In this stage, the majority of TFs in the TRN has been lost. We surmise that, in these bacteria, transcriptional regulation is exerted mainly at the structural level of DNA. In the last (extreme) stage, organisms exhibit extreme genome reduction and they no longer conserve TFs. Examples of organisms in each group are included, as well as the conserved parental classes of their gene-products according to the gene ontology classification.

From our analysis, we have developed a conceptual model of TRN evolution driven by genome reduction, which we summarize in Fig 4. Three levels of genome reduction have been already defined according to the changes organisms undergo in their gene content [18]. In the model, the early stage of network reduction is represented by lately evolved, free-living organisms, which turned into host-restricted endosymbionts and intracellular pathogens (*e*.*g*., *Sodalis glossinidius* and *Serratia symbiotica*). The genomes in this stage are characterized by the conservation of a large number of TFs that respond to environmental conditions in a manner almost similar to free-living organisms. At the advanced stage, long-term obligate endosymbionts (*e*.*g*., *Baumannia cicadellinicola and Buchnera aphidicola*) that live in more restricted habitats (generally inside insect-specialized cells) conserve a major proportion of NAPs; thus, in these organisms gene regulation may be operating mainly at a global level through nucleoid organization. In this same stage, the scenario in which all target genes regulated by a TF are lost (but not the TF itself) may happen. In such situation, we speculate that TFs assume more than one role in the cell. This is the case, for example, of BolA, which can perform both regulatory and catalytic functions, acting as a single multifunctional enzymatic system in organisms lacking two-component sensors [57]. Another example is given by HU, which apart from being involved in regulatory functions acts as a DNA organizing protein [52]. Finally, in the extreme stage, tiny-endosymbionts (*e*.*g*., *Candidatus Portiera aleyrodidarum* and *Candidatus Carsonella ruddii*) no longer retain any type of TFs, these bacteria are considered on the edge of being organelles, thus if gene regulation operates in these organisms it might be essentially at the level of intrinsic DNA topology or by physiological conditions [58], but not at the level of transcription initiation assisted by transcription factors.

## Conclusions

**Fig 5:**
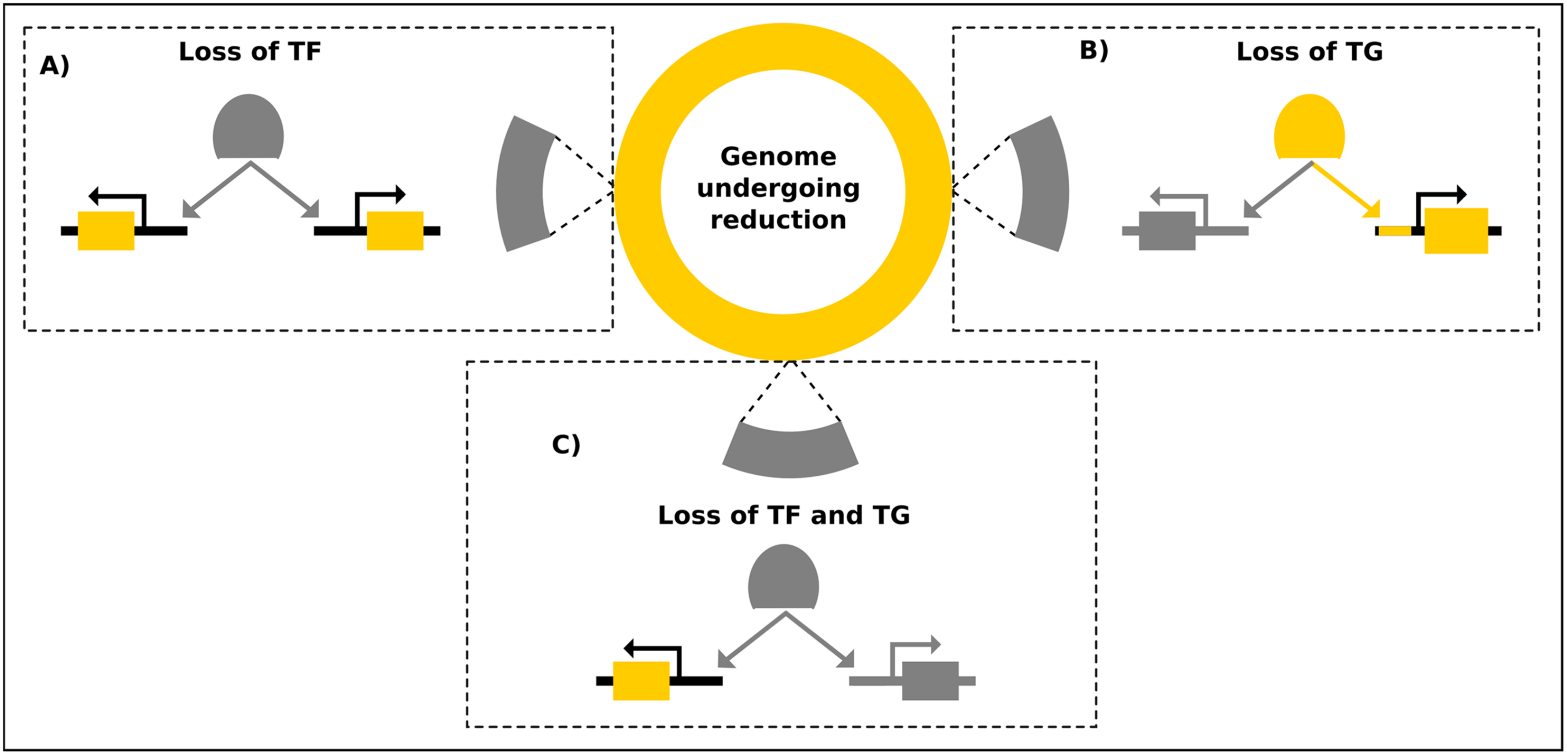
Effect of genome reduction in the transcriptional regulatory network. Behind the loss of genes (and interactions) is the genome reduction phenomenon acting as a force that directs the evolution of TRNs. Three scenarios might occur when a DNA fragment is lost in a genome: A) The fragment contains only TFs, B) The fragment contains only TGs, C) The fragment contains both TFs and TGs.

Regulatory networks in bacteria evolve throughout time following patterns of growth or reduction. That is, by means of several well-known molecular events (such as, horizontal gene transfer, point mutations, gene duplication, etc.) [12, 14], the number of TFs and target genes in a genome is increased (or decreased) leading to the acquisition (or loss) of nodes and interactions in TRNs. Earlier studies of evolution in TRNs have primarily focused on network growth and the comparison of genomes from free-living organism as the underlying methodology. The central outcomes that have arisen from these studies are that gene duplication and horizontal gene transfer are forces that direct the growth of a network [15, 16], and that TRNs of organisms change more quickly than target genes [9–11, 17]. In this work, we investigated the complementary aspect of TRN evolution under a network reduction perspective by comparing genome sequences of free-living organisms and phylogenetically-related symbionts. In these organisms, the loss of DNA fragments containing sequences coding for TFs, TGs, or both, can lead to evolution of regulatory networks (Fig 5), which have received little attention until now. We found (after reconstructing the corresponding TRNs and performing correlation analyses) that the conservation of TFs in organisms with reduced genomes is directly related to the kind of regulation they exert, with dual regulators being conserved in a larger proportion than activators and repressors in conditions of genome reduction.

Since most of bacteria exhibiting genome reduction are endosymbionts, we speculate that the lack of pressure to respond to exogenous signals makes the presence of activators dispensable in the first place. This is in agreement with our results, in which activators were the least conserved kind of regulator. As for repressors, we believe the parsimonious conditions inside a bacteriocyte make strong regulation of genes unnecessary. In agreement with our analysis, the loss of repressors followed the loss of activators. Finally, our finding that dual TFs are the most conserved regulatory elements in the transcriptional networks of these organisms might be explained in terms of their simultaneous role as global regulators and NAPs. This suggests that when the major part of the regulatory machinery is lost, gene regulation is essentially carried out at the level of structural organization of the nucleoid, pointing out the role that the conformation of DNA plays in the control of gene expression in its most intrinsic nature.

Bacteria with extreme genome reduction (*e*.*g*., with less than 170 Kbp) no longer retain known transcription factors. It remains as a mystery how these organisms regulate the expression of their genes. A possibility is that gene regulation is ultimately exerted by the physiological state of the organism (*i*.*e*., availability of ribosomes, RNA polymerases, pool of amino acids and nucleotides, etc.) [58, 59]. The investigation of this subject may have profound implications in the understanding of gene regulation at its most fundamental level and the determination of the smallest functional regulatory systems required in the design of minimal genomes, a topic of high relevance in synthetic biology.

## Supporting Information

S1 Table Transcriptional regulatory networks of the 113 bacteria analyzed here. (XLSX)

S2 Table General data of the genomes used in this work. (XLSX)

S3 Table Classification of biological function of all the orthologous genes (to *E*. *coli*) using GO. (XLSX)

S4 Table Computed probabilities from hypergeometric distribution for each type of regulators in each bacterial genome. (XLSX)

S5 Table Phylogenetically independent contrasts analyses. (XLSX)

## References

[1] Gottesman S. Bacterial regulation: global regulatory networks. Ann Rev Genet. 1984; 18: 415–41. 6099091 10.1146/annurev.ge.18.120184.002215

[2] Reznikoff WS, Siegele DA, Cowing DW, Gross CA. The regulation of transcription initiation in bacteria. Ann Rev Genet. 1985; 19: 355–387. 3936407 10.1146/annurev.ge.19.120185.002035

[3] Latchman DS. Gene Regulation. 5th ed Taylor and Francis group: 2005.

[4] Junker BH, Schreiber F. Analysis of biological networks. 1st ed Wiley Interscience; 2008.

[5] Martinez-Antonio A, Collado-Vides J. Identifying global regulators in transcriptional regulatory networks in bacteria. Curr Opin Microbiol. 2003; 6: 482–489. 14572541 10.1016/j.mib.2003.09.002

[6] Albert R. Scale-free networks in cell biology. J Cell Sci. 2005; 118: 4947–4957. 16254242 10.1242/jcs.02714

[7] Milo R, Shen-Orr S, Itzkovitz S, Kashtan N, Chklovskii D, Alon U. Network motifs: simple building blocks of complex networks. Science. 2002; 298: 824–827. 12399590 10.1126/science.298.5594.824

[8] Babu MM, Lang B, Aravind L. Methods to reconstruct and compare transcriptional regulatory networks. Methods Mol Biol. 2009; 541: 163–180. 10.1007/978-1-59745-243-4_8 19381525

[9] Babu MM, Teichmann SA, Aravind L. Evolutionary dynamics of prokaryotic transcriptional regulatory networks. J Mol Biol. 2006; 358: 614–633. 16530225 10.1016/j.jmb.2006.02.019

[10] Lozada-Chávez I, Janga SC, Collado-Vides J. Bacterial regulatory networks are extremely flexible in evolution. Nucleic Acids Res. 2006; 34: 3434–3445. 16840530 10.1093/nar/gkl423 PMC1524901

[11] Price MN, Dehal PS, Arkin AP. Orthologous transcription factors in bacteria have different functions and regulate different genes. PLOS Comput Biol. 2007; 3: 1739–1750. 17845071 10.1371/journal.pcbi.0030175 PMC1971122

[12] Wittkopp PJ, Kalay G. Cis-regulatory elements: molecular mechanisms and evolutionary processes underlying divergence. Nat Rev Genet. 2011; 13: 59–69. 10.1038/nrg3095 22143240

[13] McAdams HH, Srinivasan B, Arkin AP. The evolution of genetic regulatory systems in bacteria. Nat Rev Genet. 2004; 5: 169–178. 14970819 10.1038/nrg1292

[14] Babu MM. Structure, evolution and dynamics of transcriptional regulatory networks. Biochem Soc Trans. 2010; 38: 115–178. 10.1042/BST0381155 20863280

[15] Teichmann SA, Babu MM. Gene regulatory network growth by duplication. Nat Genet. 2004; 36: 492–496. 15107850 10.1038/ng1340

[16] Lagomarsino MC, Jona P, Bassetti B, Isambert H. Hierarchy and feedback in the evolution of the *Escherichia coli* transcription network. Proc Natl Acad Sci U.S.A. 2007; 104: 5516–5520. 17372223 10.1073/pnas.0609023104 PMC1838485

[17] Molina N, van Nimwegen E. Universal patterns of purifying selection at noncoding positions in bacteria. Genome Res. 2008; 18: 148–160. 18032729 10.1101/gr.6759507 PMC2134783

[18] McCutcheon JP, Moran NA. Extreme genome reduction in symbiotic bacteria. Nat Rev Microbiol. 2012; 10: 13–26. 10.1038/nrmicro2670 22064560

[19] Moran NA. Accelerated evolution and Muller’s rachet in endosymbiotic bacteria. Proc Natl Acad Sci U.S.A. 1996; 93: 2873–2878. 8610134 10.1073/pnas.93.7.2873 PMC39726

[20] Delaye L, Gil R, Peretó J, Latorre A, Moya A. Life With a Few Genes: A Survey on Naturally Evolved Reduced Genomes. Open Evol J. 2010; 4: 12–22.

[21] Felsenstein J. Phylogenies and the comparative method. Amer Nat. 1985; 125: 1–15.

[22] NCBI ftp server. Available: http://www.ncbi.nlm.nih.gov/FTp/.

[23] Yu H, Luscombe NM, Lu HX, Zhu X, Xia Y, Han JD, et al Annotation transfer between genomes: protein-protein interologs and protein-DNA regulogs. Genome Res. 2004; 14: 1107–1118. 15173116 10.1101/gr.1774904 PMC419789

[24] Salgado H, Peralta-Gil M, Gama-Castro S, Santos-Zavaleta A, Muñiz-Rascado L, García-Sotelo JS, et al RegulonDB v8.0: omics data sets, evolutionary conservation, regulatory phases, cross-validated gold standards and more. Nucleic Acids Res. 2013; 41: D203–D213. 10.1093/nar/gks1201 23203884 PMC3531196

[25] Moreno-Hagelsieb G, Latimer K. Choosing BLAST options for better detection of orthologs as reciprocal best hits. Bioinformatics. 2008; 24: 319–324. 18042555 10.1093/bioinformatics/btm585

[26] Keseler IM, Mackie A, Peralta-Gil M, Santos-Zavaleta A, Gama-Castro S, Bonavides-Martínez C, et al EcoCyc: fusing model organism databases with systems biology. Nucleic Acids Res. 2013; 41: D605–D612. 10.1093/nar/gks1027 23143106 PMC3531154

[27] Ashburner M, Ball CA, Blake JA, Botstein D, Butler H, Cherry JM, et al Gene ontology: tool for the unification of biology. The Gene Ontology Consortium. Nat Genet. 2000; 25: 25–29. 10802651 10.1038/75556 PMC3037419

[28] Huang da W, Sherman BT, Lempicki RA. Systematic and integrative analysis of large gene lists using DAVID Bioinformatics Resources. Nat Protoc. 2009; 4: 44–57. 10.1038/nprot.2008.211 19131956

[29] R Development core team, 2008. Available: http://www.r-project.org/.

[30] Fisher RA. Statistical methods for research workers. 5th Edition Oliver and Boyd, Edinburgh, London; 1934 pp.103–105.

[31] Chernick MR. The essentials of biostatistics for physicians, nurses and clinicians. New Jersey: John Wiley & Sons Ltd; 2011.

[32] Garland T, Harvey PH, Ives AR. Procedures for the analysis of comparative data using phylogenetically independent contrasts. Syst Biol. 1992; 41: 18–32.

[33] Garland T, Midford PE, Ives AR. An introduction to phylogenetically based statistical methods, with a new method for confidence intervals on ancestral values. Amer Zool. 1999; 39: 374–388.

[34] Garland T, Ives AR. Using the past to predict the present: confidence intervals for regression equations in phylogenetic comparative methods. Amer Zool. 2000; 155: 346–364. 10.1086/303327 10718731

[35] Maddison WP, Maddison DR. Mesquite: a modular system for evolutionary analysis (Version 3.02). 2015. Available: http://mesquiteproject.org.

[36] Ronquist F, Teslenko M, van der Mark P, Ayres DL, Darling A, Höhna S, et al MrBayes 3.2: Efficient Bayesian phylogenetic inference and model choice across a large model space. Sys Biol. 2012; 61: 539–542. 10.1093/sysbio/sys029 PMC3329765 22357727

[37] Holder M, Lewis PO. Phylogeny estimation: traditional and bayesian approaches. Nat Rev Genet. 2003; 4: 275–284. 12671658 10.1038/nrg1044

[38] Lima-Mendez G, van Helden J. The powerful law of the power law and other myths in network biology. Mol BioSyst. 2009; 5: 1482–1493. 10.1039/b908681a 20023717

[39] Galán-Vásquez E, Luna B, Martínez-Antonio A. The regulatory network of Pseudomonas aeruginosa. Microb Inform Exp. 2011; 1: 1–11. 10.1186/2042-5783-1-3 PMC3348663 22587778

[40] Alexopoulos EC. Introduction to multivariate regression analysis. Hippokratia, 2010; 14: 23–28. 21487487 PMC3049417

[41] Davis JH. Statistics for compensation: A practical guide to compensation analysis. 1st ed John Wiley & Sons, Inc; 2011.

[42] van Nimwegen E. Scaling law in the functional content of genomes. Trends Genet. 2003; 19: 479–484. 12957540 10.1016/S0168-9525(03)00203-8

[43] Pérez-Rueda E, Collado-Vides J. The repertoire of DNA-binding transcriptional regulators in *Escherichia coli* K-12. Nucleic Acids Res. 2000; 28: 1838–1847. 10734204 10.1093/nar/28.8.1838 PMC102813

[44] Balderas-Martínez YI, Savageau M, Salgado H, Pérez-Rueda E, Morett E, Collado-Vides J. Transcription factors in *Escherichia coli* prefer the *Holo* conformation. Plos One. 2013; 8: 1–9. 10.1371/journal.pone.0065723 PMC3680503 23776535

[45] Martínez-Antonio A, Janga SC, Salgado H, Collado-Vides J. Internal-sensing machinery directs the activity of the regulatory network in *Escherichia coli*. Trends Microbiol. 2006; 14: 22–27. 16311037 10.1016/j.tim.2005.11.002

[46] Struhl K. Fundamentally different logic of gene regulation in Eukaryotes and Prokaryotes. Cell. 1999; 98: 1–4. 10412974 10.1016/S0092-8674(00)80599-1

[47] Stracy M, Lesterlin C, Garza de Leon F, Uphoff S, Zawadzki P, Kapanidis AN. Live-cell superresolution microscopy reveals the organization of RNA polymerase in the bacterial nucleoid. Proc Natl Acad Sci U.S.A. 2015; 32: E4390–E4399. 10.1073/pnas.1507592112 PMC4538611 26224838

[48] Gerosa L, Kochanowski K, Heinemann M, Sauer U. Dissecting specific and global transcriptional regulation of bacterial gene expression. Mol Syst Biol. 2013, 9: 1–11. 10.1038/msb.2013.14 PMC3658269 23591774

[49] Alon U. Network motifs: theory and experimental approaches. Nat Rev Genet. 2007; 8: 450–461. 17510665 10.1038/nrg2102

[50] Madar D, Dekel E, Bren A, Alon U. Negative auto-regulation increases the input dynamic-range of the arabinose system of *Escherichia coli*. BMC Syst Biol. 2011; 5: 1–9. 21749723 10.1186/1752-0509-5-111 PMC3163201

[51] Klumpp S, Zhang Z, Hwa T. Growth rate-dependent global effects on gene expression in bacteria. Cell. 2009, 139: 1366–1375. 10.1016/j.cell.2009.12.001 20064380 PMC2818994

[52] Dillon SC, Dorman CJ. Bacterial nucleoid-associated proteins, nucleoid structure and gene expression. Nat Rev Microbiol. 2010; 8: 185–195. 10.1038/nrmicro2261 20140026

[53] Huynen MA, Spronk CA, Gabaldon T, Snel B. Combining data from genomes, Y2H and 3D structure indicates that BolA is a reductase interacting with a glutaredoxin. FEBS Lett. 2005; 579: 591–596. 15670813 10.1016/j.febslet.2004.11.111

[54] Savageau MA. Design of molecular control mechanisms and the demand for gene expression. Proc Natl Acad Sci U.S.A. 1977; 74: 5647–5651. 271992 10.1073/pnas.74.12.5647 PMC431845

[55] Savageau MA. Demand theory of gene regulation. II. Quantitative application to the lactose and maltose operons of *Escherichia coli*. Genetics, 1998; 149: 1677–1691. 9691028 10.1093/genetics/149.4.1677 PMC1460280

[56] Moran NA, Bennett GM. The tiniest tiny genomes. Annu Rev Microbiol. 2014; 68: 195–215. 10.1146/annurev-micro-091213-112901 24995872

[57] Briza L, Calevro F, Charles H. Genomic analysis of the regulatory elements and links with intrinsic DNA structural properties in the shrunken genome of *Buchnera*. BMC Genomics. 2013; 14: 1–15. 23375088 10.1186/1471-2164-14-73 PMC3571970

[58] Scott M, Hwa T. Bacterial growth laws and their applications. Curr Opin Biotechnol. 2011; 22: 559–565. 10.1016/j.copbio.2011.04.014 21592775 PMC3152618

[59] Berthoumieux S, de Jong H, Baptist G, Pinel C, Ranquet C, Ropers D, et al Shared control of gene expression in bacteria by transcription factors and global physiology of the cell. Mol Syst Biol. 2013; 9 (634): 1–11. 10.1038/msb.2012.70 PMC3564261 23340840

